# Growing Disparities in Patient-Provider Messaging: Trend Analysis Before and After Supportive Policy

**DOI:** 10.2196/14976

**Published:** 2019-10-07

**Authors:** Nicole Senft, Evan Butler, Jordan Everson

**Affiliations:** 1 Department of Medicine Vanderbilt University Medical Center Nashville, TN United States; 2 Department of Health Policy Vanderbilt University School of Medicine Nashville, TN United States

**Keywords:** eHealth, policy, communication, secure messaging, disparities, socioeconomic factors, inequality

## Abstract

**Background:**

Public policy introduced since 2011 has supported provider adoption of electronic medical records (EMRs) and patient-provider messaging, primarily through financial incentives. It is unclear how disparities in patients’ use of incentivized electronic health (eHealth) tools, like patient-provider messaging, have changed over time relative to disparities in use of eHealth tools that were not directly incentivized.

**Objective:**

This study examines trends in eHealth disparities before and after the introduction of US federal financial incentives. We compare rates of patient-provider messaging, which was directly incentivized, with rates of looking for health information on the Web, which was not directly incentivized.

**Methods:**

We used nationally representative Health Information National Trends Survey data from 2003 to 2018 (N=37,300) to describe disparities in patient-provider messaging and looking for health information on the Web. We first reported the percentage of individuals across education and racial and ethnic groups who reported using these tools in each survey year and compared changes in unadjusted disparities during preincentive (2003-2011) and postincentive (2011-2018) periods. Using multivariable linear probability models, we then examined adjusted effects of education and race and ethnicity in 3 periods—preincentive (2003-2005), early incentive (2011-2013), and postincentive (2017-2018)—controlling for sociodemographic and health factors. In the postincentive period, an additional model tested whether internet adoption, provider access, or providers’ use of EMRs explained disparities.

**Results:**

From 2003 to 2018, overall rates of provider messaging increased from 4% to 36%. The gap in provider messaging between the highest and lowest education groups increased by 10 percentage points preincentive (*P*<.001) and 22 additional points postincentive (*P*<.001). The gap between Hispanics and non-Hispanic whites increased by 3.2 points preincentive (*P*=.42) and 11 additional points postincentive (*P*=.01). Trends for blacks resembled those for Hispanics, whereas trends for Asians resembled those for non-Hispanic whites. In contrast, education-based disparities in looking for health information on the Web (which was not directly incentivized) did not significantly change in preincentive or postincentive periods, whereas racial disparities narrowed by 15 percentage points preincentive (*P*=.008) and did not significantly change postincentive. After adjusting for other sociodemographic and health factors, observed associations were similar to unadjusted associations, though smaller in magnitude. Including internet adoption, provider access, and providers’ use of EMRs in the postincentive model attenuated, but did not eliminate, education-based disparities in provider messaging and looking for health information on the Web. Racial and ethnic disparities were no longer statistically significant in adjusted models.

**Conclusions:**

Disparities in provider messaging widened over time, particularly following federal financial incentives. Meanwhile, disparities in looking for health information on the Web remained stable or narrowed. Incentives may have disproportionately benefited socioeconomically advantaged groups. Future policy could address disparities by incentivizing providers treating these populations to adopt messaging capabilities and encouraging patients’ use of messaging.

## Introduction

### Background

The use of Web-based tools to access health information and health services (electronic health, eHealth) has increased along with the widespread dissemination of the internet [1,2]. However, since 2000, observers have noted a persistent *digital divide* in the diffusion of eHealth tools across segments of the US population, with members of traditionally underserved groups (lower socioeconomic status, older, and racial and ethnic minorities) being less likely to engage in many eHealth activities [3-7]. Over the past decade, several federally supported initiatives, such as regional extension centers and state cooperatives, were implemented to accelerate the spread of some eHealth tools, including Web-based communication between patients and providers. The largest supportive policy was direct financial incentives for *Meaningful Use* (MU) of eHealth records, which began in 2011 and was redesigned as the Promoting Interoperability program within the Merit-Based Incentive Payment System in 2017. Other eHealth tools, such as those facilitating access to Web-based health information that is independent of health care providers, have spread without direct health policy intervention. It is not clear how the digital divide in the use of eHealth tools financially incentivized by federal policy has changed since the enactment of supportive policy compared with the digital divide in the use of eHealth tools that were not directly incentivized by policy makers.

Trends through 2014 suggested that people with higher incomes and education remained more likely to use the internet to look up health information and to communicate with their providers, with disparities based on race sometimes becoming statistically nonsignificant in adjusted models [2,8,9]. Throughout this period, group differences in internet and home computer access at least partly explained disparities in eHealth use [5,10]. Rapid dissemination of the internet, encouraged in part by public support through programs such as the Broadband Opportunities Technology Program, have reduced racial and socioeconomic disparities in internet access and use in recent years [11]. The digital divide in overall eHealth use may have similarly decreased, although these trends have not been examined in recent data. It is also possible that disparities in eHealth use persist, perhaps because of differences in eHealth literacy or perceived benefits and concerns of using eHealth [4,12,13]. Furthermore, disparities in use of eHealth tools to communicate with providers may be uniquely driven by differences in access to health care providers [14]. Individuals who have not seen any provider are not likely to communicate with one on the Web [15], and racial and socioeconomic disparities in access to providers persist despite increased insurance coverage following the Affordable Care Act [12].

Beyond differences in provider access, disparities may be driven by uneven adoption of functional electronic medical records (EMRs) among providers [16,17]. The MU program was designed as an all-or-nothing incentive in which providers either qualified in a year and received a substantial payment or did not receive any incentive payment. MU criteria became progressively more difficult: In 2011, providers were encouraged to develop the ability to send patients reminder messages to receive incentive payments. By 2015, providers were required to send secure messages to at least 5% of unique patients to receive incentives. Only 62% of physicians were able to attest to MU by 2016, and high-resource physician offices (which are more likely to treat high-resource patients) were more likely to attest to MU than low-resource physician offices [16,18,19]. Therefore, it is possible that incentives encouraging electronic communication between patients and providers have unintentionally widened disparities in its adoption and use by inadequately addressing access to providers and, in particular, to providers with highly usable EMRs who encourage eHealth communication [20].

### Objectives

In this study, we characterized trends in disparities in 2 eHealth technologies from 2003 to 2018 to examine whether the digital divide persists and whether financial incentives contributed to narrowing or widening that divide. Specifically, we described the use of 1 incentivized technology (communicating with providers via messaging) and 1 technology that was not directly incentivized (looking for health information on the Web) over time and across socioeconomic strata and racial groups. Finally, we investigated whether internet adoption, health care access, and providers’ use of EMRs explain the digital divide in recent years.

## Methods

### Data

We used data from the National Cancer Institute’s Health Information National Trends Survey (HINTS), a cross-sectional, nationally representative survey of noninstitutionalized adults in the United States. HINTS was developed to monitor changes in health communication and information technology. We used data from 8 iterations of HINTS, administered in 2003 (HINTS 1), 2005 (HINTS 2), 2008 (HINTS 3), 2011 (HINTS 4, cycle 1), 2013 (HINTS 4, cycle 3), 2015 (HINTS-FDA), 2017 (HINTS 5, cycle 1), and 2018 (HINTS 5, cycle 2). Detailed information on each iteration’s sampling methodology, data collection, and response rates are published by the National Cancer Institute [21].

### Population

The full sample included 37,300 individual-year responses from 2003 to 2018. As different questions were included in different years, we excluded observations with missing data on an analysis-by-analysis basis, as described in the Analysis section.

### Dependent Variables: Electronic Health Use

Use of provider messaging was assessed by asking participants whether, in the past 12 months, they had “used e-mail or the Internet to communicate with a doctor or a doctor’s office.” This item was not asked in 2015 (HINTS-FDA). Looking for health information on the Web was assessed by asking participants whether, in the past 12 months, they had used the internet to “look for health or medical information for yourself.” This item was not asked in 2008 (HINTS 3). Response options for both items were *yes/no*. Before 2017, only individuals who reported using the internet (n=20,445) were asked to respond to these questions. Noninternet users (n=9994) in these survey years, who were not asked these items, were recoded as responding “no” to them, as it is reasonable to assume noninternet users were not using the internet to message their providers or look up health information.

### Independent Variables: Sociodemographic and Health-Related Variables

We defined racial and ethnic groups and socioeconomic strata using 2 measures included on all years of HINTS data. To measure race and ethnicity, we defined 6 different racial and ethnic groups: Hispanic, non-Hispanic white, black, Asian, other (American Indian/Native Alaskan/Native Hawaiian/Pacific Islander), and multiracial. We defined socioeconomic strata by the level of education, categorized as 4 levels in all years: less than high school, high school graduate or General Education Diploma, some college or technical school, and college graduate or greater.

We included several additional demographic and health-related variables in multivariable models. Demographic variables included household income (<US $20,000, US $20,000- US $34,999, US $35,000- US $49,999, US $50,000- US $74,999, US $75,000- US $99,999, and > US $100,000), sex (male and female), age (18-34 years, 35-49 years, 50-64 years, 65-74 years, and >75 years), and marital status (married, living as married/member of an unmarried couple, divorced, widowed, separated, and single). Measurement of household income in 2003 differed from other survey years: the lowest income group was less than US $25,000 and the highest was greater than or equal to US $75,000. Health-related variables included health insurance coverage (yes or no) and general health (excellent, very good, good, fair, and poor).

### Explanatory Factors

We explored 3 potential explanations for observed health disparities in 2017 to 2018: internet adoption, access to a health care provider, and having a provider that uses an EMR. Internet adoption was assessed by a single item: “Do you ever go on-line to access the Internet or World Wide Web, or to send and receive e-mail?” (yes/no). Access to a health care provider was assessed by the item, “In the past 12 months, not counting times you went to an emergency room, how many times did you go to a doctor, nurse, or other health professional to get care for yourself?” This item was dichotomized to reflect those who had versus those who had not visited a health care provider in the past year. Finally, provider use of an EMR was defined using the item, “Do any of your doctors or other health care providers maintain your medical records in a computerized system?” (yes/no).

### Analysis

We first described the overall rates of provider messaging and looking for health information on the Web over time by plotting the percentage of all individuals who reported using these eHealth tools in each survey year. Analyses included all nonmissing responses to provider messaging (n=32,742, average responses per year=4677; minimum=2905, maximum=7612) and looking for health information on the Web (n=28,663, average responses per year=4090; minimum=2885, maximum=6350).

Next, we described disparities over time in provider messaging and looking for health information on the Web by plotting the percentage of individuals across levels of education and by racial and ethnic group who reported using these tools in each survey year. Multimedia Appendix 1 additionally plots the use of these tools across income levels.

We then compared the unadjusted change in magnitude of disparities in eHealth use by education and race and ethnicity during the preincentive period (2003 vs 2011) and postincentive period (2011 vs 2018). Linear regression models included an indicator for year, education level or race and ethnicity, and the interaction between year and education or race and ethnicity. We added and subtracted regression coefficients using linear combinations to generate mean differences and SEs of differences across groups and years. Two racial and ethnic groups (Native American/Pacific Islander and Multiracial) were excluded from this analysis because they did not have at least 100 observations in each survey year. Respondents were excluded from analyses if they were missing data on education (3%) or race (7%).

We generated adjusted estimates of the associations of education and race and ethnicity with provider messaging and looking for health information on the Web using multivariable linear probability models. Analyses included both education and race and ethnicity and were adjusted for household income, sex, age, marital status, insurance coverage, and general health. To examine changing adjusted associations over time, we replicated models in 3 separate periods: before major public investment (2003-2005), the first years of public support (2011-2013), and recent years (2017-2018). In constructing these periods, we excluded the 2008 and 2015 survey years in which only 1 dependent variable was included in the survey instrument. Finally, in 2017 to 2018, an additional regression model added internet adoption, access to health care providers, and providers’ use of an EMR to the multivariable model described above to test whether these factors explained the disparities that persisted in 2017 to 2018. Only cases with complete data were used in analyses; sample sizes for each regression analysis can be found in Table 1. Survey weights were applied in all analyses, which were conducted using Stata 15 MP by StataCorp, College Station, TX.

**Table 1:** The associations of education and race with electronic health use in preincentive (2003-2005), early incentive (2011-2013), and postincentive (2017-2018) periods. Analyses are adjusted for individuals’ income, age, gender, marital status, insurance status, and health status. Linear probability models were generated using complete case analyses. Unweighted sample sizes for each model are provided in parentheses. Survey weights were used to generate means reflective of the US population. *P* values were created using jackknife SEs. Full regression results and SEs are available in Multimedia Appendix 2.

Independent variables			Provider messaging				Looking for health information on the Web			
			2003-2005 (n=9954)	2011-2013 (n=5292)	2017-2018A (n=5305)	2017-2018B^a^ (n=5294)	2003-2005 (n=9950)	2011-2013 (n=5333)	2017-2018A (n=5317)	2017-2018B (n=5306)
**Education level (Reference: Less than high school)**										
	High school graduate		−0.001	−0.01	0.05	0.03	0.08^b^	0.09	0.07	0.02
	Some college		0.03^b^	0.09^b^	0.14^b^	0.11^c^	0.22^b^	0.27^b^	0.21^b^	0.13
	College graduate		0.06^b^	0.13^b^	0.24^b^	0.18^c^	0.34^b^	0.35^b^	0.28^b^	0.19^b^
**Race (Reference: non-Hispanic white)**										
	Hispanic		−0.02	−0.002	−0.05^a^	−0.03	−0.11^b^	−0.05	−0.05	−0.01
	Black		−0.00	−0.01	0.02	−0.03	−0.07^b^	−0.02	−0.03	−0.01
	Asian		−0.02	0.05	0.03	−0.06	−0.02	−0.05	−0.03	0.01
	Multiple races selected		0.01	0.03	0.02	−0.03	0.04	−0.14	0.09^c^	0.08
	Other		0.01	−0.003	−0.01	−0.06	−0.10^c^	−0.22^c^	−0.26	−0.26
**Explanatory factors**										
	Uses internet		—^d^	—	—	0.14^b^	—	—	—	0.39^b^
	Seen physician in previous 12 months		—	—	—	0.11^b^	—	—	—	0.10^b^
	**Physician uses EMR^e^ (Reference: No)**									
		Yes	—	—	—	0.20^b^	—	—	—	0.05
		Do not know	—	—	—	0.03	—	—	—	0.02
Constant			0.01	0.05	0.06	−0.21	0.21	0.48	0.52	0.19

^a^An additional model (2017-2018B) included potential explanatory factors: internet adoption, provider access, and providers’ use of EMRs.

^b^*P*<.01.

^c^*P*<.05.

^d^Not applicable.

^d^EMR: electronic medical record.

## Results

### Overall Trends

#### Provider Messaging

**Figure 1:**
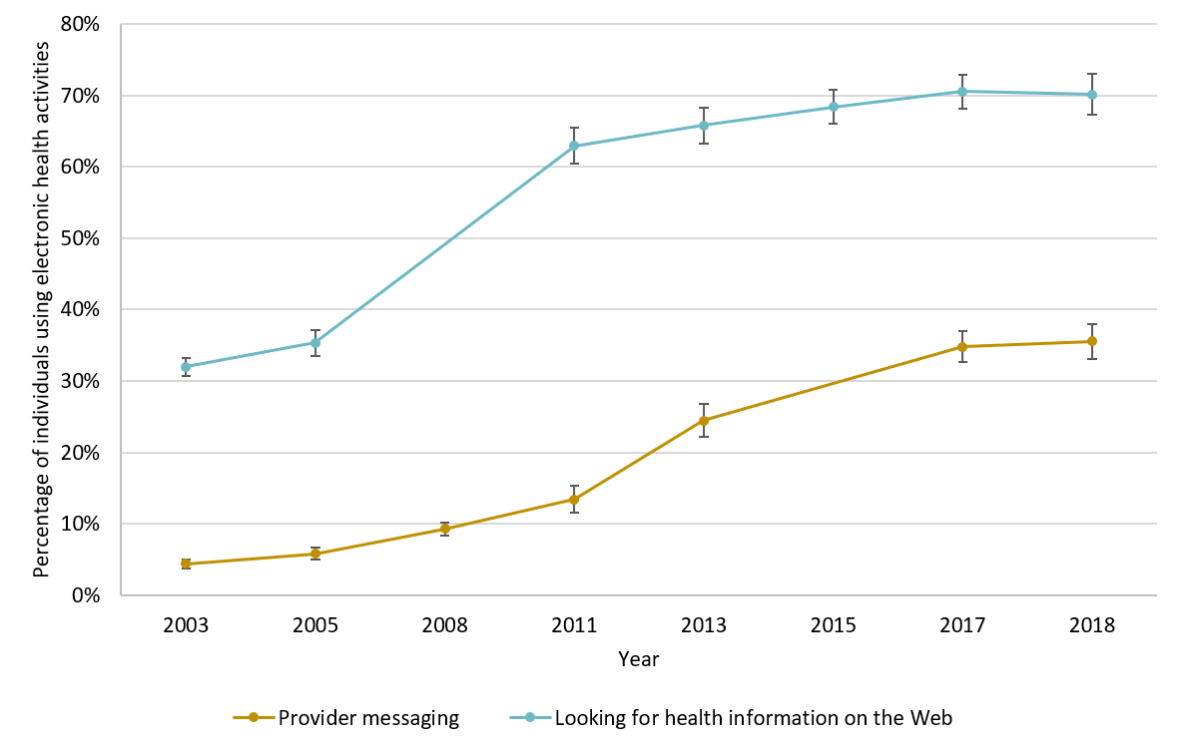
Prevalence of electronic health use, 2003 to 2018. The sample for provider messaging includes 32,742 total responses (average 4677 per year), and the sample for looking for health information on the Web includes 28,663 total responses (average 4090 per year). Survey weights were used to generate means reflective of the US population. Bars represent 95% CIs generated using jackknife SEs.

The population-weighted percentage of individuals using provider messaging increased by 32 percentage points, from 4.4% in 2003 to 36% in 2018 (Figure 1). Growth was relatively slow during the first years of the study period, increasing by 9.0 percentage points (from 4.4% to 13%) between 2003 and 2011. Growth was more rapid in later years, after the enactment of relevant public policies starting in 2011, increasing by 22 percentage points between 2011 and 2018 (from 13% to 36%).

#### Looking for Health Information on the Web

The percentage of individuals that reported looking for health information on the Web increased by 38 percentage points, from 32% in 2003 to 70% in 2018. In contrast to provider messaging, growth was rapid during the first years of the study period and slowed in later years. Between 2003 and 2011, the percentage increased by 31 percentage points (from 32% to 63%) and then increased by only 7.2 percentage points between 2011 and 2018 (from 63% to 70%).

### Trends in Disparities

#### Education

**Figure 2:**
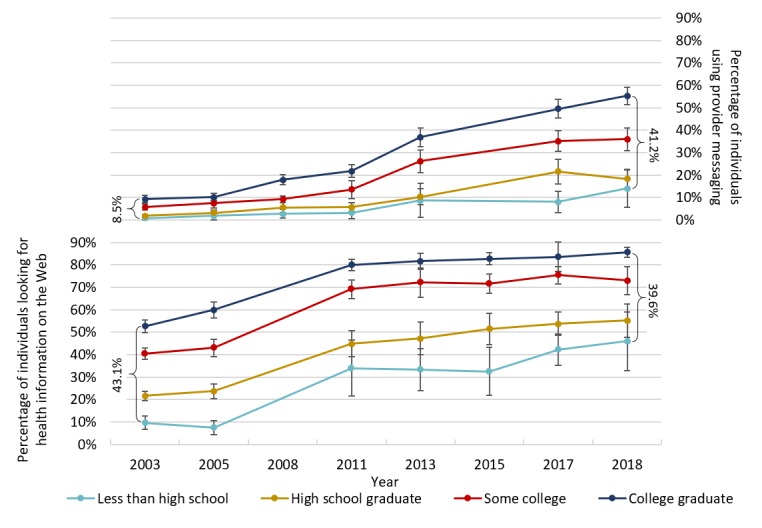
Electronic health use by education level, 2003 to 2018. The sample for provider messaging includes 31,672 total responses, and the sample for looking for health information on the Web includes 27,860 total responses. Survey weights were used to generate means reflective of the US population. Bars represent 95% CIs generated using jackknife SEs. Brackets represent the difference in prevalence between the highest and lowest education groups in the first and last years of the analysis. Of the overall respondents, 3% were not included in this analysis because they were missing information on education.

Reported use of provider messaging increased across all education groups between 2003 and 2018 (Figure 2, top panel), although growth was slower among individuals with lower levels of education. Among individuals who did not complete high school, rates of provider messaging increased by only 14 percentage points, with most of this increase (11 points) occurring after 2011. Over the same period, there was a 46 percentage point increase among college graduates, again with most of the change (34 points) occurring after 2011. The gap between the highest and lowest education groups increased by 10 percentage points between 2003 and 2011 (*P*<.001) and an additional 22 percentage points between 2011 and 2018 (*P*<.001).

Reported use of the internet to look for health information also increased across all education groups (Figure 2, bottom panel). In contrast to provider messaging, the increase in looking for health information on the Web was similar across education levels. From 2003 to 2018, reported rates increased by 36 percentage points among individuals who did not complete high school, compared with 33 percentage points among those with a college degree. For both groups, the majority of this increase occurred in the preincentive period; from 2003 to 2011, rates increased by 27 percentage points among individuals who did not complete high school and 24 percentage points among college graduates. Therefore, the gap between the highest and lowest education groups increased by 2.9 percentage points between 2003 and 2011 (*P*=.69) and then narrowed by 6.2 percentage points between 2011 and 2018 (*P*=.50).

#### Race and Ethnicity

**Figure 3:**
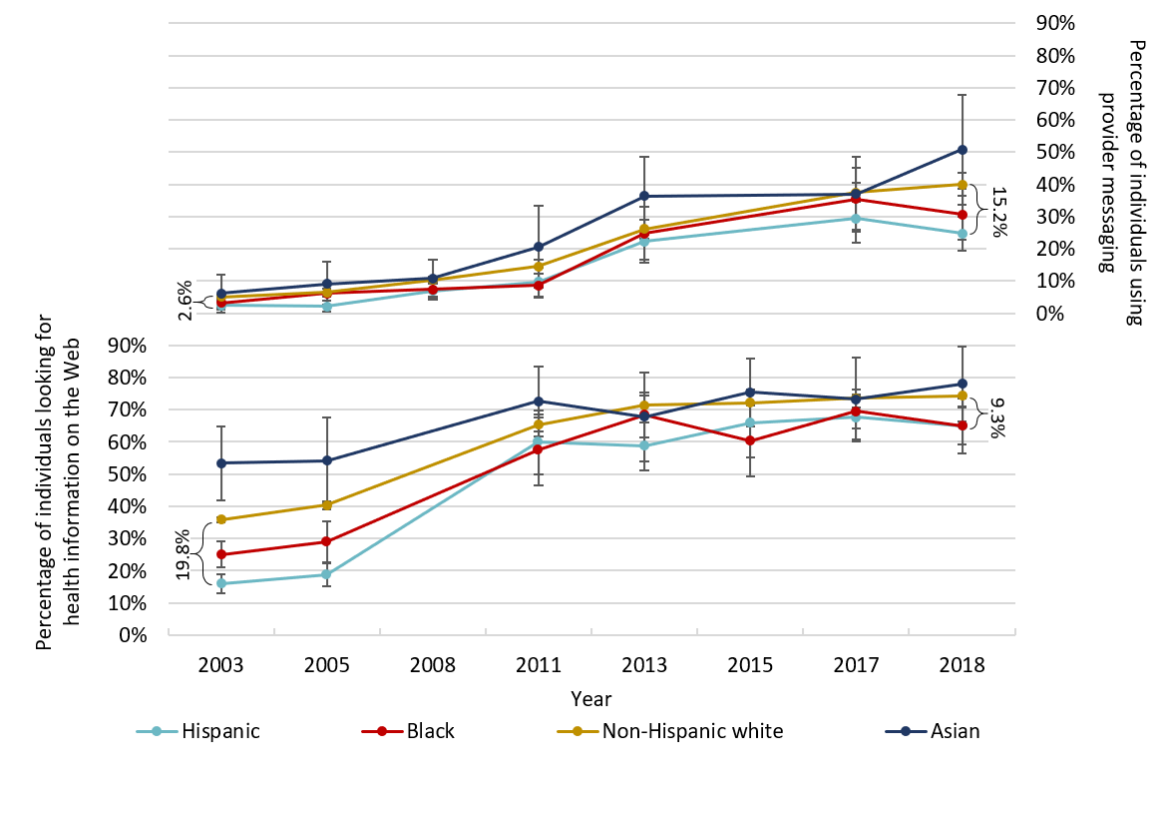
Electronic health use by race and ethnicity. The sample for provider messaging includes 29,484 total responses, and the sample for looking for health information on the Web includes 25,638 total responses. Survey weights were used to generate means reflective of the US population. Bars represent 95% CIs generated using SEs. Brackets represent the difference in prevalence between Hispanic and non-Hispanic white respondents in the first and last years of the analysis. Other and multiracial categories were excluded from this analysis because they did not have at least 100 observations for each sample year. Furthermore, 7% of total respondents were excluded because they did not indicate a race.

Use of provider messaging increased across all racial and ethnic groups between 2003 and 2018 (Figure 3, top panel), although the growth was slower among traditionally underserved groups, as was the case with education groups. Among Hispanics, who reported messaging providers least often, the reported rates of provider messaging increased by 22 percentage points, the smallest increase of any group. Meanwhile, among non-Hispanic whites, the reported rates increased by 35 percentage points. As with education, the majority of these increases occurred in the postincentive period: between 2011 and 2018, rates of provider messaging increased by 15 percentage points among Hispanics and by 25 percentage points among non-Hispanic whites. The gap in use of provider messaging between Hispanics and non-Hispanic whites increased by only 2.1 points between 2003 and 2011 (*P*=.42) but widened an additional 11 points between 2011 and 2018 (*P*=.01). Trends for blacks closely followed those for Hispanics, whereas trends for Asians more closely resembled those for non-Hispanic whites.

The rates of looking for health information on the Web also increased across all racial and ethnic groups. However, unlike provider messaging, the reported rates of looking for health information on the Web increased most quickly among traditionally underserved racial groups. Among Hispanics, the reported rates increased by 49 percentage points between 2003 and 2018, the greatest increase of any group. Among non-Hispanic whites, the rates increased by 38 percentage points. Paralleling trends across education levels, the majority of this increase occurred in the preincentive period, with rates increasing by 44 percentage points for Hispanics and 30 percentage points for non-Hispanic whites between 2003 and 2011. Therefore, the gap between Hispanic and non-Hispanic whites narrowed by 15 points between 2003 and 2011 (*P*=.008) and then widened by 3.8 points between 2011 and 2018 (*P*=.53).

### Adjusted Associations

#### Education

The magnitude of the adjusted association between education and provider messaging increased over time but was smaller than the unadjusted differences presented above (Table 1). In 2003 to 2005, college graduates were 6.2 percentage points more likely to use provider messaging than those with less than a high school education (*P*<.001), whereas in 2017 to 2018, college graduates were 24 percentage points more likely to use provider messaging than those with less than a high school education (*P*<.001). In comparison, the adjusted association between education and looking for health information on the Web grew less strong over time. In 2003 to 2005, college graduates were 34 percentage points more likely to look for health information on the Web than those with less than a high school education (*P*<.001). By 2017 to 2018, that difference decreased to 28 percentage points (*P*<.001).

#### Race and Ethnicity

As with education, the associations between race and ethnicity and provider messaging increased over time, whereas the associations with looking up health information on the Web lessened. In 2003 to 2005, non-Hispanic whites were 2.0 percentage points more likely to use provider messaging than Hispanics (*P*=.07), compared with 5.4 percentage points in 2017 to 2018 (*P*=.05). In contrast, in 2003 to 2005, non-Hispanic whites were 11 percentage points more likely than Hispanics to look for health information on the Web (*P*<.001). By 2017 to 2018, the difference decreased to 5.2 percentage points (*P*=.06).

#### Explanatory Factors

In 2017 to 2018, internet adoption and access to health care providers were strongly associated with both provider messaging and looking for health information on the Web. Physician use of an EMR was associated with a 20 percentage point increase in the likelihood of provider messaging (*P*<.001) but was not associated with looking up health information on the Web. Including these variables in the model attenuated, but did not eliminate, the associations between education and provider messaging or looking for health information on the Web. Disparities across racial and ethnic groups in provider messaging and looking for health information on the Web were no longer statistically significant when accounting for these explanatory factors.

## Discussion

### Principal Findings

In nationally representative data from 2003 to 2018, both provider messaging and looking up health information on the Web became more common. However, the *digital divide* in the use of financially incentivized, provider-focused eHealth (provider messaging) widened, whereas the divide in eHealth that is independent of providers and policy-based incentives (looking up health information on the Web) stayed the same across education levels and narrowed across racial and ethnic groups. For all groups, the rates of provider messaging grew more rapidly in the years following the introduction of federal financial incentives, whereas the rates of looking up information on the Web plateaued. Disparities that persisted in 2017 to 2018 were only partially explained by differences in internet adoption, health care access, or provider use of an EMR. These findings indicate that federal incentives may have accelerated growth in the technologies they targeted across all groups, but they may have disproportionately impacted growth among white, well-educated, and wealthier individuals.

Existing theories and models of patient adoption of health technologies suggest that persistent disparities in health technologies stem from systematic differences at the patient, provider, and system levels [22-24]. Financial incentives directed at providers address provider participation in secure messaging, but they may have failed to address several other provider-level factors that differentially impact groups of patients. Although our data included a measure of whether providers maintained an EMR, they did not include measures of whether providers offered secure messaging to patients. Providers serving underresourced communities may not offer secure messaging to patients because they are too busy, are not comfortable with eHealth tools themselves, hold beliefs or biases that these groups of patients are unlikely to use or benefit from eHealth tools, or are less able or willing to change workflows to facilitate the use of sometimes cumbersome messaging tools [25-28]. One key issue is that EMRs vary in quality, and adopting poorer-quality EMRs may make messaging time consuming, difficult, and poorly integrated into existing clinical workflows [29-31]. Public policies, including MU, may have exacerbated the differences in EMR quality. High-resource practices, which often serve wealthier patient groups, were more likely to use advance EMRs, attest to MU, and receive payments [32-35]. In contrast, lower-resource practices may have either viewed the MU criteria as too challenging or have adopted systems that were just good enough to facilitate MU attestation. The challenges of working with poorer-quality EMRs are likely made more difficult by the fact that practices serving low-resource patients tend to have especially limited time for each patient visit [36].

Beyond these important provider-level considerations, evidence indicates that several differences in patient-level factors, such as health literacy, eHealth literacy, attitudes toward Web-based health information, and social norms, also contribute to continued disparities in eHealth use [4,5,10,12,13]. Some related mechanisms may be unique to the growing disparities in the use of provider messaging. For example, evidence suggests that there are barriers to high-quality interpersonal communication between patients who are racial and/or ethnic minorities, have lower levels of education, or have lower incomes and their providers [37-39]. Poorer interpersonal communication may discourage patients from communicating with physicians outside of the clinic setting through eHealth. Finally, although the spread of the internet has narrowed the digital divide [11], African Americans, Hispanics, and low-income individuals are more likely than white or wealthier Americans to rely on smartphones for their internet connections, leading to unreliable access, especially for those who reach their maximum monthly data allotments or have to cancel or suspend phone services because of financial hardship [40-42]. The measure of internet adoption used in this research did not capture these kinds of disparities in access that may impede patients’ ability to engage in health technologies. Underresourced patients are also more likely to share devices or use publicly available devices, perhaps sparking concerns about the privacy and security of sharing their health information over the internet. Indeed, a previous study has shown that African Americans are more concerned about health information security than whites and that these concerns predict engagement in eHealth more strongly for African Americans’ than they do for whites [13].

As supplements to newer policy efforts under the Merit-Based Incentive Payment System, public policy should consider ways to overcome barriers that are particularly likely to impede provider messaging with patients in lower-resource settings. These might include further development of team-based approaches that move some of the burden of messaging from physicians to other professionals, provider education around strategies to maximize benefit from messaging, and support for provider outreach programs specifically targeting disadvantaged patients. Policy makers may also need to complement provider-facing initiatives with other programs designed to increase digital inclusion. For example, a previous study has shown that lacking internet access in one’s neighborhood is a major factor associated with patient portal use [43], suggesting a need for further efforts aimed at increasing reliable, secure access to the internet. Other efforts could target patient-level barriers to adoption, including general skills training to improve proficiency of internet use and targeted training to orient patients to portal use and/or secure messaging. Policy makers might also consider how to provide incentives for smaller EMR vendors to enhance the usability of patient messaging platforms to close the *advanced use* gap between providers in high- versus low-resource settings [17]. Although our data are from the United States, other countries may similarly find that without careful design, the benefits of information technology investment and financial incentives disproportionately benefit some groups over others.

We presented both unadjusted (Figures 1-3) and adjusted (Table 1) associations between demographic variables and eHealth use because of the closely intertwined nature of race, education, and income. Multivariate modeling approaches that attempt to isolate the independent effects of each may obscure important relationships by controlling for parts of the causal pathway. In these data, lower rates of eHealth use among some racial and ethnic minority groups are mostly accounted for by the inclusion of education, income, and other predictors in adjusted models. This suggests that policies addressing differences by education and income, for example, by increasing eHealth literacy or incentivizing providers’ use of secure messaging in low-resource settings, may be most impactful while also decreasing racial disparities.

We report absolute differences in eHealth use between groups, although some previous literature [1,12] has focused on relative differences. Given the rapidly changing rates of use across all groups during this period, absolute differences are more readily interpretable. Still, both absolute and relative differences indicate that the divide in provider messaging is worse relative to the divide in the use of the internet to look up health information. We find that the digital divide in looking for health information on the Web has stayed constant, whereas the divide in provider messaging has grown. In relative terms, the divide in looking up health information on the Web has decreased (eg, from eight-fold in 2005 to two-fold in 2018 across education groups), whereas the divide in provider messaging has stayed fairly constant (eg, from five-fold to four-fold).

### Limitations

Our study is subject to a number of limitations. HINTS is a cross-sectional survey that does not allow for longitudinal analysis of change in individuals over time but rather changes by group characteristics; therefore, selection bias in specific survey years may influence our findings. Furthermore, similar to all survey data, HINTS data may be subject to nonresponse or self-reporting bias. Our analysis does not support causal inference: although we highlight that changing rates of eHealth use parallel enactment of public policy related to provider messaging, specifically federal financial incentives to providers, we cannot definitively state that the incentives caused these changes. Similarly, as we have discussed, it is likely that unobserved mediating variables are more proximal causes of the digital divide than the demographic variables measured here. Finally, our analysis was constrained to only 2 eHealth activities that were asked in most years of HINTS. These activities are representative of provider-focused, incentivized eHealth and of eHealth that is independent of providers and not directly incentivized [44], but they differ in other ways as well. For example, looking up health information on the Web was more common in the baseline year than provider messaging, which may in part explain the tapering increases in use in later years. Using additional measures of eHealth activities would bolster our inference that the relationship with federal financial incentives influenced diverging rates of use of each activity, but those measures are not available.

### Conclusions

Using recent, nationally representative data on individuals’ use of Web-based tools, we identified a growing digital divide in the rate of messaging with health care providers relative to looking for health information on the Web. This indicates that although federal financial incentive initiatives were successful at increasing patient-provider messaging across all groups, they may have also disproportionately benefited socioeconomically advantaged groups. Moving forward, policy makers should consider how redesigned policy initiatives and new policies might reduce disparities in the use of tools intended to facilitate communication between patients and providers.

## Appendix

Multimedia Appendix 1 Electronic health use by income, 2003-2018. The sample for provider messaging includes 28,238 total responses, and the sample for looking for health information online includes 25,080 total responses. Survey weights were used to generate means reflective of the US population. Bars represent 95% CIs, generated using jackknife SEs. Income was coded differently in 2003, the lowest group was less than US $25k and the highest was above US $75k, these groups were therefore excluded from the figure in that survey year.

Multimedia Appendix 2 Results of multivariable logistic regression models predicting provider messaging and looking for health information on the Web in 3 separate periods: before major public investment (2003-2005), the first years of public support (2011-2013), and recent years (2017-2018). Furthermore, 2 additional models included 3 variables to test potential explanations for remaining disparities in electronic health use in 2017 to 2018: whether respondents were internet users, whether they had seen a doctor in the past year, and whether their health care provider maintained electronic medical records. Analyses were adjusted for individuals’ income, age, gender, marital status, insurance status, and health status. Linear probability models were generated using complete case analyses. Survey weights were used to generate means reflective of the US population. P values were created using jackknife SEs. SEs are presented in parentheses.

## Abbreviations

AHRQ: Agency for Healthcare Research and Quality
eHealth: electronic health
EMR: electronic medical record
HINTS: Health Information National Trends Survey
MU: meaningful use
PCORI: Patient-Centered Outcomes Research Institute

## References

[1] Hong YA, Cho J (2017). Has the digital health divide widened? Trends of health-related internet use among older adults from 2003 to 2011. J Gerontol B Psychol Sci Soc Sci.

[2] Prestin A, Vieux SN, Chou WS (2015). Is online health activity alive and well or flatlining? Findings from 10 years of the health information national trends survey. J Health Commun.

[3] Powell JA, Darvell M, Gray JA (2003). The doctor, the patient and the world-wide web: how the internet is changing healthcare. J R Soc Med.

[4] Levy H, Janke AT, Langa KM (2015). Health literacy and the digital divide among older Americans. J Gen Intern Med.

[5] Nguyen A, Mosadeghi S, Almario CV (2017). Persistent digital divide in access to and use of the internet as a resource for health information: results from a California population-based study. Int J Med Inform.

[6] Collins SA, Yoon S, Rockoff ML, Nocenti D, Bakken S (2016). Digital divide and information needs for improving family support among the poor and underserved. Health Informatics J.

[7] Anthony DL, Campos-Castillo C, Lim PS (2018). Who isn't using patient portals and why? Evidence and implications from a national sample of US adults. Health Aff (Millwood).

[8] Huerta TR, Walker DM, Johnson T, Ford EW (2016). A time series analysis of cancer-related information seeking: hints from the health information national trends survey (HINTS) 2003-2014. J Health Commun.

[9] Greenberg AJ, Serrano KJ, Thai CL, Blake KD, Moser RP, Hesse BW, Ahern DK (2017). Public use of electronic personal health information: measuring progress of the Healthy People 2020 objectives. Health Policy Technol.

[10] Chesser A, Burke A, Reyes J, Rohrberg T (2016). Navigating the digital divide: a systematic review of ehealth literacy in underserved populations in the United States. Inform Health Soc Care.

[11] (2019). Pew Research Center.

[12] Graetz I, Gordon N, Fung V, Hamity C, Reed ME (2016). The digital divide and patient portals: internet access explained differences in patient portal use for secure messaging by age, race, and income. Med Care.

[13] Senft N, Abrams J, Katz A, Barnes C, Charbonneau DH, Beebe-Dimmer JL, Zhang K, Eaton T, Heath E, Thompson HS (2019). eHealth activity among African American and white cancer survivors: a new application of theory. Health Commun.

[14] Tarver WL, Menser T, Hesse BW, Johnson TJ, Beckjord E, Ford EW, Huerta TR (2018). Growth dynamics of patient-provider internet communication: trend analysis using the health information national trends survey (2003 to 2013). J Med Internet Res.

[15] Sudore RL, Mehta KM, Simonsick EM, Harris TB, Newman AB, Satterfield S, Rosano C, Rooks RN, Rubin SM, Ayonayon HN, Yaffe K (2006). Limited literacy in older people and disparities in health and healthcare access. J Am Geriatr Soc.

[16] Heisey-Grove D, King JA (2017). Physician and practice-level drivers and disparities around meaningful use progress. Health Serv Res.

[17] Adler-Milstein J, Holmgren AJ, Kralovec P, Worzala C, Searcy T, Patel V (2017). Electronic health record adoption in US hospitals: the emergence of a digital 'advanced use' divide. J Am Med Inform Assoc.

[18] Sandefer RH, Marc DT, Kleeberg P (2015). Meaningful use attestations among us hospitals: the growing rural-urban divide. Perspect Health Inf Manag.

[19] Wieringa S, Greenhalgh T (2015). 10 years of mindlines: a systematic review and commentary. Implement Sci.

[20] Ohno-Machado L (2017). Understanding and mitigating the digital divide in health care. J Am Med Inform Assoc.

[21] Health Information National Trends Survey | HINTS.

[22] Venkatesh V, Morris MG, Davis GB, Davis FD (2003). User acceptance of information technology: toward a unified view. MIS Q.

[23] McGinn CA, Grenier S, Duplantie J, Shaw N, Sicotte C, Mathieu L, Leduc Y, Légaré F, Gagnon M (2011). Comparison of user groups' perspectives of barriers and facilitators to implementing electronic health records: a systematic review. BMC Med.

[24] Palacholla RS, Fischer N, Coleman A, Agboola S, Kirley K, Felsted J, Katz C, Lloyd S, Jethwani K (2019). Provider- and patient-related barriers to and facilitators of digital health technology adoption for hypertension management: scoping review. JMIR Cardio.

[25] Miller Jr DP, Latulipe C, Melius KA, Quandt SA, Arcury TA (2016). Primary care providers' views of patient portals: interview study of perceived benefits and consequences. J Med Internet Res.

[26] Wolcott V, Agarwal R, Nelson DA (2017). Is provider secure messaging associated with patient messaging behavior? Evidence from the US army. J Med Internet Res.

[27] Heyworth L, Clark J, Marcello TB, Paquin AM, Stewart M, Archambeault C, Simon SR (2013). Aligning medication reconciliation and secure messaging: qualitative study of primary care providers' perspectives. J Med Internet Res.

[28] Thompson MJ, Reilly JD, Valdez RS (2016). Work system barriers to patient, provider, and caregiver use of personal health records: a systematic review. Appl Ergon.

[29] Carayon P, Wetterneck TB, Alyousef B, Brown RL, Cartmill RS, McGuire K, Hoonakker PL, Slagle J, van Roy KS, Walker JM, Weinger MB, Xie A, Wood KE (2015). Impact of electronic health record technology on the work and workflow of physicians in the intensive care unit. Int J Med Inform.

[30] Howard J, Clark EC, Friedman A, Crosson JC, Pellerano M, Crabtree BF, Karsh B, Jaen CR, Bell DS, Cohen DJ (2013). Electronic health record impact on work burden in small, unaffiliated, community-based primary care practices. J Gen Intern Med.

[31] Friedberg MW, Chen PG, van Busum KR, Aunon F, Pham C, Caloyeras J, Mattke S, Pitchforth E, Quigley DD, Brook RH, Crosson FJ, Tutty M (2014). Factors affecting physician professional satisfaction and their implications for patient care, health systems, and health policy. Rand Health Q.

[32] Green LA, Potworowski G, Day A, May-Gentile R, Vibbert D, Maki B, Kiesel L (2015). Sustaining 'meaningful use' of health information technology in low-resource practices. Ann Fam Med.

[33] Samuel CA (2014). Area-level factors associated with electronic health record adoption and meaningful use in the Regional Extension Center Program. J Am Med Inform Assoc.

[34] Bishop TF, Press MJ, Mendelsohn JL, Casalino LP (2013). Electronic communication improves access, but barriers to its widespread adoption remain. Health Aff (Millwood).

[35] Kruse CS, Kristof C, Jones B, Mitchell E, Martinez A (2016). Barriers to electronic health record adoption: a systematic literature review. J Med Syst.

[36] Reschovsky JD, O'Malley AS (2008). Do primary care physicians treating minority patients report problems delivering high-quality care?. Health Aff (Millwood).

[37] Penner LA, Eggly S, Griggs JJ, Underwood III W, Orom H, Albrecht TL (2012). Life-threatening disparities: the treatment of black and white cancer patients. J Soc Issues.

[38] Eggly S, Hamel LM, Foster TS, Albrecht TL, Chapman R, Harper FW, Thompson H, Griggs JJ, Gonzalez R, Berry-Bobovski L, Tkatch R, Simon M, Shields A, Gadgeel S, Loutfi R, Ali H, Wollner I, Penner LA (2017). Randomized trial of a question prompt list to increase patient active participation during interactions with black patients and their oncologists. Patient Educ Couns.

[39] Johnson RL, Roter D, Powe NR, Cooper LA (2004). Patient race/ethnicity and quality of patient-physician communication during medical visits. Am J Public Health.

[40] Anderson M, Kumar M (2019). Pew Research Center.

[41] Perrin A, Turner E (2019). Pew Research Center.

[42] Smith A (2015). Pew Research Center.

[43] Perzynski AT, Roach MJ, Shick S, Callahan B, Gunzler D, Cebul R, Kaelber DC, Huml A, Thornton JD, Einstadter D (2017). Patient portals and broadband internet inequality. J Am Med Inform Assoc.

[44] Senft N, Everson J (2018). eHealth engagement as a response to negative healthcare experiences: cross-sectional survey analysis. J Med Internet Res.

