# Supplementary material for: Growing Disparities in Patient-Provider Messaging: Trend Analysis Before and After Supportive Policy
Source: J Med Internet Res. 2019 Oct 7;21(10):e14976. doi: 10.2196/14976 (PMC6803888; doi:10.2196/14976)

Appendix 1. eHealth use by income, 2003-2018. The sample for provider messaging includes 28,238 total responses, and the sample for looking for health information online includes 25,080 total responses. Survey weights were used to generate means reflective of the US population. Bars represent 95% confidence intervals, generated using jackknife standard errors. Income was coded differently in 2003, the lowest group was less than $25k and the highest was above $75k, these groups were therefore excluded from the figure in that survey year.

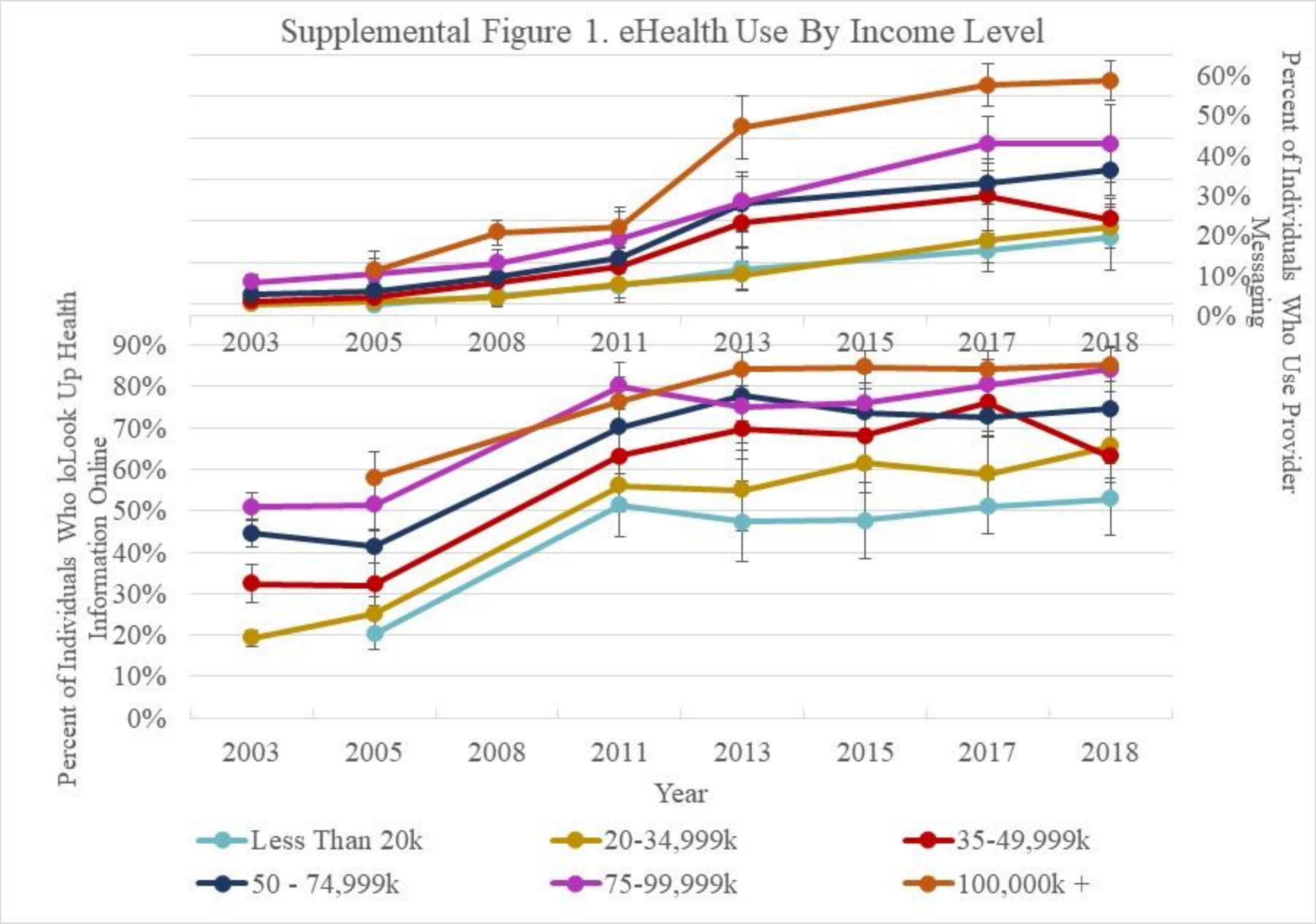

Supplement: Multimedia Appendix 1 [file jmir_v21i10e14976_app1.pdf]
