# Supplementary material for: Growing Disparities in Patient-Provider Messaging: Trend Analysis Before and After Supportive Policy
Source: J Med Internet Res. 2019 Oct 7;21(10):e14976. doi: 10.2196/14976 (PMC6803888; doi:10.2196/14976)
Supplement: Multimedia Appendix 2 [file jmir_v21i10e14976_app2.pdf]

**Multimedia Appendix 2. Full results of multivariable regressions.** Results of multivariable logistic regression models predicting provider messaging and looking for health information online in three separate periods: before major public investment (2003-2005), the first years of public support (2011-2013) and recent years (2017-2018). Two additional models included three variables to test potential explanations for remaining disparities in eHealth use in 2017-2018: whether respondents were internet users, had seen a doctor in the past year, and whether their healthcare provider maintained electronic medical records. Analyses are adjusted for individuals' income, age, gender, marital status, insurance status and health status. Linear probability models generated using complete case analyses. Survey weights were used to generate means reflective of the US population. P-values created using jackknife standard errors. Standard errors presented in parentheses.

| **Variables** | **Provider Messaging** | | | | **Looking for Health Information Online** | | | |
|---|---|---|---|---|---|---|---|---|
| | **2003-2005** | **2011-2013** | **2017-2018A** | **2017-2018B** | **2003-2005** | **2011-2013** | **2017-2018A** | **2017-2018B** |
| | | | | | | | | |
| **Education Level (Ref: Less than High School Graduate)** | | | | | | | | |
| **High School Graduate** | -0.001 | -0.006 | 0.050 | 0.033 | 0.080** | 0.086 | 0.066 | 0.020 |
| | (0.008) | (0.026) | (0.040) | (0.041) | (0.017) | (0.064) | (0.061) | (0.068) |
| **Some College** | 0.033** | 0.090** | 0.143** | 0.107* | 0.222** | 0.268** | 0.211** | 0.131 |
| | (0.011) | (0.031) | (0.044) | (0.047) | (0.019) | (0.061) | (0.057) | (0.067) |
| **College Graduate** | 0.062** | 0.133** | 0.236** | 0.183** | 0.341** | 0.349** | 0.284** | 0.187** |
| | (0.013) | (0.035) | (0.043) | (0.048) | (0.024) | (0.059) | (0.051) | (0.062) |
| **Race (Ref: non-Hispanic White)** | | | | | | | | |
| **Hispanic** | -0.020 | -0.002 | -0.054* | -0.030 | -0.110** | -0.051 | -0.052 | -0.011 |
| | (0.011) | (0.027) | (0.026) | (0.027) | (0.019) | (0.038) | (0.027) | (0.029) |
| **Black** | -0.002 | -0.009 | 0.024 | 0.029 | -0.073** | -0.018 | -0.029 | -0.014 |
| | (0.012) | (0.028) | (0.028) | (0.028) | (0.020) | (0.029) | (0.027) | (0.027) |
| **Other** | 0.014 | -0.003 | -0.098 | -0.061 | -0.100* | -0.221* | -0.258 | -0.262 |
| | (0.024) | (0.064) | (0.108) | (0.112) | (0.047) | (0.108) | (0.255) | (0.287) |
| **Asian** | -0.021 | 0.052 | 0.034 | 0.064 | -0.019 | -0.048 | -0.033 | 0.008 |
| | (0.026) | (0.045) | (0.070) | (0.062) | (0.042) | (0.050) | (0.036) | (0.035) |
| **Multiple Races Selected** | 0.006 | 0.034 | 0.022 | 0.029 | 0.035 | -0.137 | 0.085* | 0.082* |
| | (0.026) | (0.048) | (0.063) | (0.061) | (0.038) | (0.082) | (0.038) | (0.032) |
| **Income Level (Ref: Less Than $20,000)** | | | | | | | | |
| **$20,000 - $34,999** | 0.003 | 0.007 | -0.007 | -0.021 | -0.006 | 0.050 | 0.057 | 0.022 |
| | (0.009) | (0.023) | (0.036) | (0.033) | (0.020) | (0.046) | (0.045) | (0.039) |
| **$35,000 - $49,999** | 0.000 | 0.072* | 0.043 | 0.020 | 0.005 | 0.135** | 0.101** | 0.048 |
| | (0.010) | (0.033) | (0.040) | (0.040) | (0.025) | (0.041) | (0.034) | (0.034) |
| **$50,000 - $74,999** | 0.011 | 0.095** | 0.101** | 0.070* | 0.081** | 0.178** | 0.114** | 0.049 |
| | (0.010) | (0.029) | (0.037) | (0.034) | (0.023) | (0.042) | (0.041) | (0.041) |
| **$75,000 - $99,999** | 0.033* | 0.108** | 0.142** | 0.099* | 0.107** | 0.184** | 0.168** | 0.092* |
| | (0.015) | (0.034) | (0.045) | (0.042) | (0.030) | (0.051) | (0.040) | (0.041) |
| **$100k +** | 0.050** | 0.189** | 0.283** | 0.235** | 0.168** | 0.189** | 0.161** | 0.084 |
| | (0.017) | (0.039) | (0.047) | (0.046) | (0.038) | (0.045) | (0.040) | (0.046) |
| **Age (Ref: 18-34)** | | | | | | | | |
| **35-49** | -0.014 | -0.002 | -0.004 | -0.002 | -0.077** | -0.074** | -0.056 | -0.034 |
| | (0.009) | (0.028) | (0.033) | (0.030) | (0.015) | (0.028) | (0.030) | (0.028) |
| **50-64** | -0.010 | -0.016 | -0.042 | -0.040 | -0.135** | -0.152** | -0.125** | -0.093** |
| | (0.009) | (0.030) | (0.033) | (0.033) | (0.017) | (0.022) | (0.029) | (0.029) |

| | | | | | | | | |
|---|---|---|---|---|---|---|---|---|
| **65-74** | -0.036** | -0.062 | -0.064 | -0.061 | -0.247** | -0.246** | -0.201** | -0.135** |
| | (0.010) | (0.032) | (0.032) | (0.034) | (0.021) | (0.030) | (0.036) | (0.036) |
| **75+** | -0.038** | -0.026 | -0.139** | -0.124** | -0.309** | -0.406** | -0.381** | -0.254** |
| | (0.011) | (0.040) | (0.042) | (0.046) | (0.020) | (0.040) | (0.047) | (0.042) |
| **Marital Status (Ref: Married)** | | | | | | | | |
| **Living as Married** | 0.016 | 0.007 | -0.022 | -0.006 | 0.007 | -0.056 | 0.015 | 0.015 |
| | (0.015) | (0.037) | (0.043) | (0.044) | (0.027) | (0.054) | (0.049) | (0.043) |
| **Divorced** | 0.004 | 0.022 | -0.033 | -0.023 | 0.017 | -0.005 | -0.005 | 0.008 |
| | (0.008) | (0.024) | (0.025) | (0.025) | (0.016) | (0.028) | (0.033) | (0.028) |
| **Widowed** | 0.007 | -0.057* | -0.036 | -0.005 | -0.035 | -0.090* | -0.142** | -0.088* |
| | (0.011) | (0.025) | (0.038) | (0.036) | (0.018) | (0.040) | (0.042) | (0.037) |
| **Separated** | 0.007 | 0.059 | -0.091* | -0.100* | -0.027 | -0.029 | -0.133 | -0.141 |
| | (0.015) | (0.060) | (0.043) | (0.049) | (0.040) | (0.054) | (0.086) | (0.128) |
| **Single** | 0.023 | 0.002 | -0.032 | -0.007 | 0.032 | -0.001 | -0.026 | -0.010 |
| | (0.012) | (0.027) | (0.024) | (0.024) | (0.022) | (0.032) | (0.030) | (0.033) |
| **Gender (Ref: Female)** | | | | | | | | |
| **Male** | -0.006 | -0.058** | -0.074** | -0.044* | -0.110** | -0.087** | -0.059** | -0.042* |
| | (0.006) | (0.019) | (0.019) | (0.019) | (0.014) | (0.020) | (0.019) | (0.018) |
| **Insurance Status (Ref: Uninsured)** | | | | | | | | |
| **Insured** | 0.008 | 0.067** | 0.085 | 0.020 | 0.063** | -0.020 | -0.026 | -0.071 |
| | (0.010) | (0.023) | (0.055) | (0.048) | (0.020) | (0.032) | (0.041) | (0.044) |
| **Health Status (Ref: Excellent)** | | | | | | | | |
| **Very Good** | 0.005 | -0.059 | 0.062 | 0.047 | 0.021 | 0.012 | 0.099* | 0.090* |
| | (0.010) | (0.036) | (0.033) | (0.030) | (0.019) | (0.029) | (0.045) | (0.041) |
| **Good** | 0.016 | 0.004 | 0.064* | 0.059* | 0.050* | 0.055* | 0.127** | 0.122** |
| | (0.010) | (0.038) | (0.028) | (0.027) | (0.019) | (0.025) | (0.045) | (0.045) |
| **Fair** | 0.008 | -0.016 | 0.040 | 0.031 | 0.043* | 0.071 | 0.132** | 0.132** |
| | (0.009) | (0.047) | (0.040) | (0.039) | (0.021) | (0.038) | (0.045) | (0.046) |
| **Poor** | 0.021 | 0.007 | 0.126* | 0.104 | 0.082* | 0.092 | 0.075 | 0.051 |
| | (0.012) | (0.059) | (0.062) | (0.064) | (0.035) | (0.064) | (0.080) | (0.080) |
| **Explanatory Factors** | | | | | | | | |
| **Uses Internet (Ref: No)** | | | | 0.144** | | | | 0.386** |
| | | | | (0.037) | | | | (0.041) |
| **Seen Physician in Prior 12 Mos. (Ref: No)** | | | | 0.110** | | | | 0.100** |
| | | | | (0.025) | | | | (0.032) |
| **Physician Uses EHR (Ref: No)** | | | | | | | | |
| **Yes** | | | | 0.199** | | | | 0.045 |
| | | | | (0.051) | | | | (0.053) |
| **Don't Know** | | | | 0.032 | | | | 0.017 |
| | | | | (0.054) | | | | (0.063) |
| **Constant** | 0.013 | 0.046 | 0.063 | -0.211* | 0.209** | 0.476** | 0.524** | 0.187 |
| | (0.016) | (0.048) | (0.078) | (0.084) | (0.033) | (0.074) | (0.078) | (0.101) |
| | | | | | | | | |
| **Observations** | 9,954 | 5,292 | 5,305 | 5,294 | 9,950 | 5,333 | 5,317 | 5,306 |
| **R-squared** | 0.032 | 0.091 | 0.146 | 0.191 | 0.195 | 0.198 | 0.180 | 0.264 |

**** p<0.01, * p<0.05**
